# Dental Luminary

**Published:** 1869-11

**Authors:** 


### DENTAL LUMINARY.

A new edition of this little work for popular reading has just been issued, revised, and enlarged by the addition of ten pages of new matter. This consists of directions in the use of artificial teeth, a short article on the indications of good fillings, the code of ethics of the American Dental Association, and the law to regulate the practice of Dentistry in the State ot Ohio.

This work we regard as the best thing upon the whole, for popular reading, upon the teeth, and the relations of the Dentist to the public, that we have seen.

The code of ethics, and the law, will certainly attract the attention and awaken the interest of every reader. And we would simply suggest to our professional brethren that no more simple and effective method of conveying a knowledge of the status of our profession to the people, could be employed than the distribution of this or a similar work.

We will willingly supply it to any one who may wish it for distribution. It contains thirty-four pages, 16mo, and cost $5,50 per hundred in plain paper cover, and $7,00 per hundred in black enameled paper, gilt, with card upon cover. T.

